# Dexibuprofen Biodegradable Nanoparticles: One Step Closer towards a Better Ocular Interaction Study

**DOI:** 10.3390/nano10040720

**Published:** 2020-04-10

**Authors:** Elena Sánchez-López, Gerard Esteruelas, Alba Ortiz, Marta Espina, Josefina Prat, Montserrat Muñoz, Amanda Cano, Ana Cristina Calpena, Miren Ettcheto, Antoni Camins, Zaid Alsafi, Eliana B. Souto, Maria Luisa García, Montserrat Pujol

**Affiliations:** 1 Department of Pharmacy, Pharmaceutical Technology and Physical Chemistry, Faculty of Pharmacy, University of Barcelona, 08028 Barcelona, Spain; gesterna7@alumnes.ub.edu (G.E.); albaortiz@ub.edu (A.O.); m.espina@ub.edu (M.E.); jprat@ub.edu (J.P.); mmunozjuncosa@ub.edu (M.M.); acanofernandez@ub.edu (A.C.); anacalpena@ub.edu (A.C.C.); marisagarcia@ub.edu (M.L.G.); mopujol@ub.edu (M.P.); 2 Institute of Nanoscience and Nanotechnology (IN2UB), Universitat de Barcelona, 08028 Barcelona, Spain; 3 Center for Biomedical Research in Neurodegenerative Diseases Network (CIBERNED), Carlos III Health Institute, 28031 Madrid, Spain; mirenettcheto@ub.ed (M.E.); camins@ub.edu (A.C.); 4 Department of Pharmacology, Toxicology and Therapeutic Chemistry, Faculty of Pharmacy, University of Barcelona, 08028 Barcelona, Spain; 5 Glaucoma and Retinal Neurodegeneration Research Visual Neuroscience, UCL Institute of Ophthalmology, Bath Street, London EC1V 9EL, UK; zaid.alsafi.14@ucl.ac.uk; 6 Department of Pharmaceutical Technology, Faculty of Pharmacy, University of Coimbra, Pólo das Ciências da Saúde, Azinhaga de Santa Comba, 3000-548 Coimbra, Portugal; ebsouto@ff.uc.pt; 7 CEB—Centre of Biological Engineering, University of Minho, Campus de Gualtar, 4710-057 Braga, Portugal

**Keywords:** dexibuprofen, drug delivery system, nanoparticles, PLGA

## Abstract

Ocular inflammation is one of the most prevalent diseases in ophthalmology, which can affect various parts of the eye or the surrounding tissues. Non-steroidal anti-inflammatory drugs (NSAIDs) such as ibuprofen, are commonly used to treat ocular inflammation in the form of eye-drops. However, their bioavailability in ocular tissues is very low (less than 5%). Therefore, drug delivery systems such as biodegradable polymeric PLGA nanoparticles constitute a suitable alternative to topical eye administration, as they can improve ocular bioavailability and simultaneously reduce drug induced side effects. Moreover, their prolonged drug release can enhance patient treatment adherence as they require fewer administrations. Therefore, several formulations of PLGA based nanoparticles encapsulating dexibuprofen (active enantiomer of Ibuprofen) were prepared using the solvent displacement method employing different surfactants. The formulations have been characterized and their interactions with a customized lipid corneal membrane model were studied. Ex vivo permeation through ocular tissues and in vivo anti-inflammatory efficacy have also been studied.

## 1. Introduction

Ibuprofen is a widely used analgesic, antipyretic and anti-inflammatory drug [1]. Dexibuprofen (DXI) is the single pharmacologically effective enantiomer of racemic ibuprofen [2]. In the dose ratio of 1:0.5 (racemic ibuprofen vs. DXI), several pain models show that DXI is as effective, or superior to racemic ibuprofen. In addition, DXI has demonstrated comparable efficacy to diclofenac, naproxen and celecoxib [2]. Therefore, it constitutes a suitable candidate for ocular drug delivery in order to treat ocular inflammation. DXI has shown favorable tolerability when compared to other NSAIDs but still shows some adverse effects associated with this family. Moreover, eye drops often require frequent instillations, due to the rapid and extensive pre-corneal loss caused by drainage through the nasolacrimal duct, and by the non-productive absorption after corneal permeation and blinking [3]. Therefore, in order to reduce DXI adverse effects and extend its release by decreasing the number of instillations required, DXI loaded nanoparticles administered as eye drops may represent a viable solution to the problem.

Nanocarriers are particularly attractive as they provide protection to the drug, increase drug efficacy, permeate physiological barriers and decrease toxicity [1]. In this sense, biodegradable nanoparticles made of poly-D,L-lactic-co-glycolic acid (PLGA) may represent a suitable candidate to encapsulate DXI and deliver it slowly into the target tissue [4]. In addition, PLGA has been approved by the Food and Drug Administration and is one of the most successful biodegradable polymers [3,4,5,6]. Moreover, PEG has been widely used to reduce the clearance of PLGA nanoparticles (NPs) and it has been demonstrated that PEG coatings shield the surface from aggregation, opsonization, phagocytosis and prolong nanoparticles systemic circulation time [5]. Poly(ethylene glycol) (PEG) is a non-ionic, hydrophilic, polyether synthesized by polymerization of the monomer ethylene glycol and can be obtained in a wide range of molecular weights [7,8].

For this reason, several % of PEG have been studied and compared with PLGA NPs. Additionally, the role of various surfactants commonly used for ocular drug delivery in the formation, characterization and efficacy of PLGA and PLGA PEG NPs have been studied. In this sense, widely used non-ionic surfactants such as Lutrol F68 (Poloxamer 188), Tween 80^®^ and polyvinyl alcohol (PVA) have been studied [9].

In addition, the development of corneal membrane models able to mimic the interactions between the cornea and the drug delivery systems is still an unmet medical need. Therefore, a corneal membrane model has been developed mimicking the lipid structure of the corneal surface and the interactions between the different DXI NPs. Hence, one of the aims of this study will be to develop an in vitro corneal membrane model that is suitable to study the interactions of drugs and drug delivery systems with their surface. The results will be correlated with the in vitro drug release of DXI from the NPs and with the ex vivo and in vivo therapeutic efficacy. In vitro and in vivo ocular tolerance has also been assured for all the formulations prior to in vivo experiments.

## 2. Materials and Methods

### 2.1. Preparation of Polymeric Nanoparticles

PLGA nanoparticles (NPs) containing DXI were prepared by using the solvent displacement method [10,11]. In summary, 90 mg of PLGA (Boehringuer Ingelheim^®^, Ingelheim am Rhein, Germany) and 5 mg of dexibuprofen (Amadis Chemical^®^, Hangzhou, Zhejiang, China) were weighed and dissolved in 5 mL of acetone. The organic solution was added dropwise into 10 mL of an aqueous surfactant solution at pH 3.5 under magnetic stirring. Following this, the acetone was evaporated under reduced pressure. Various optimized concentrations of surfactants were used (PVA 0.5%, Tween 80^®^ 0.3% and Lutrol F68 (1%). NPs prepared using PVA were centrifuged at 15,000 rpm for 30 min in order to remove excess of PVA. Empty PLGA NPs were prepared using the same protocol but without dexibuprofen.

### 2.2. Characterization of Formulations

#### 2.2.1. Particle Size, Zeta Potential and Polydispersity (PI)

Particle size and polydispersity index (PI) were determined by dynamic light scattering using a Zetasizer nano ZS (Malvern instruments, IESMAT, Alcobendas, Madrid, Spain) at 25 °C and scattering angle of 90 °C. Zeta potential (ZP) was determined by electrophoresis laser-Doppler using the same instrument. Samples were previously diluted 1:10 (v/v) in water and the assays were carried out in triplicate.

#### 2.2.2. Entrapment Efficiency (EE)

The amount of DXI entrapped in the NPs was calculated indirectly using/Equation (1)/[1]. DXI NPs were diluted 1:100 (*v*/*v*) in water and filtration-centrifugation at 14,000 rpm for 30 min was carried out. The amount of free DXI in the supernatant was determined by high-performance liquid chromatography (HPLC).

(1) $$\mathrm{EE}\;(\%)=\frac{Total\;amount\;of\;DXI-Free\;DXI}{Total\;amount\;of\;DXI}\times 100$$

HPLC quantification was carried out 220 nm using a Kromasil^®^C18 (5 µm, 150 mm × 4.6 mm with a mobile phase 80:20 (methanol: orthophosphoric acid 0.05%) [11].

#### 2.2.3. Transmission Electron Microscopy

Transmission electron microscopy (TEM) was used to investigate the morphology of the DXI NPs on a Jeol 1010 (Jeol USA, Dearborn Road, Peabody, MA 01960, USA). Copper grids were activated with UV light and samples were diluted (1:10) and placed on the grid surface to visualize the particles. Samples were previously subjected to negative staining with uranyl acetate (2%) [12].

#### 2.2.4. Interaction Studies

Differential scanning calorimetry (DSC) analysis was performed using a DSC 823e System Mettler-Toledo, Barcelona, Spain. A pan with indium (purity ≥99.95%; Fluka, Switzerland) was used to check the calibration of the calorimetric system. An empty pan served as a reference. The DSC measurements were carried out in all DXI NPs formulations using a heating ramp from 25 to 105 °C at 10 °C/min in a nitrogen atmosphere. Data was evaluated using the Mettler STARe V 9.01 dB software (Mettler-Toledo, Barcelona, Spain) [5].

X-ray spectroscopy (XRD) was used to analyze the amorphous or crystalline state of the samples (centrifuged DXI NPs). Samples were sandwiched between 3.6 μm polyester films and exposed to CuKα radiation (45 kV, 40 mA, λ = 1.5418 Å) in the range (2θ) of 2–60° with a step size of 0.026° and a measuring time of 200 s per step [13].

Fourier transform infrared (FTIR) spectra of DXI NPs were obtained using a Thermo Scientific Nicolet iZ10 with an ATR diamond and DTGS detector (Barcelona, Spain) [12].

### 2.3. In Vitro Release Study

In order to study the in vitro drug release of DXI from the drug delivery systems, a bulk-equilibrium reverse dialysis bag technique was applied [11]. This technique is based on the dispersion of the colloidal suspension in the dialysis medium accomplishing sink conditions.

The release medium was composed of a buffer solution (PBS 0.1 M, pH 7.4) and 14 dialysis sacs containing 0.5 mL of PBS were previously immersed. A volume of 7 mL of free DXI or DXI loaded NPs were added to 136 mL of the dissolution medium. The assay was carried out by triplicate comparing the formulations. Release kinetic experiments were performed at a fixed temperature of 32 °C (ocular surface temperature) under constant magnetic stirring (*n* = 6/group). At predetermined time intervals, dialysis sacs were withdrawn from the stirred release solution and the volume was replaced by 0.5 mL of PBS. The content of the sacs at each time point was evaluated and data were adjusted to the most common kinetic models [14].

### 2.4. Sterilization Using Gamma Radiation

DXI NPs were sterilized using gamma irradiation using ^60^Co at a dose of 25 kGy (Aragogamma, Barcelona, Spain) [15]. According to the European Pharmacopoeia, 25 kGy represents the adequate absorbed dose for the purpose of sterilizing pharmaceutical products when bioburden is not known.

Furthermore, it is considered a standard γ-irradiation dose recommended for terminal sterilization of medical products that maintain a valid sterility assurance level of 10^−6^ [16].

### 2.5. In Vitro Ocular Irritation Assay: HET-CAM

In vitro ocular irritation assay was carried out in order to measure the potential irritation properties of the developed formulations. This assay was based on the INVITTOX 15 protocol [17].

During the test, the direct observation of the irritant effects (bleeding, vasoconstriction and coagulation) in the chorioallantoic membrane (CAM) of an embryonated egg (10 days) induced by application of 300 µL of the studied formulation, was performed during the first 5 min [18]. In the experimental procedure, fertilized and incubated eggs during 10 days were used. These eggs (from the farm G.A.L.L.S.A, Tarragona) were placed in the incubator at controlled temperature (37.8 °C) and humidity (50–60%). A series of controls were performed: SDS 1% (positive control for slow irritation), 0.1 N NaOH (positive control for fast irritation) and NaCl 0.9% (negative the injury occurred (*n* = 6/group).

Data were analyzed as the Ocular irritation index (OII) mean ± SD was calculated by applying Equation (2). OII can be grouped into four categories [11].

(2) $$\mathrm{OII}=\frac{5\left(301-H\right)}{300}+\frac{7\left(301-V\right)}{300}+\frac{9\left(301-C\right)}{300}$$

Meaning, H hemorrhage, V vasoconstriction and C coagulation phenomena.

### 2.6. In Vivo Ocular Irritation Assay

In vivo ocular tolerance was measured using primary eye irritation test of Draize et al. using New Zealand albino rabbits of 2.5 kg from San Bernardo farm (Navarra) [19]. This test was performed according to the Ethical Committee for Animal Experimentation of the UB and current legislation (Decret 214/97, Gencat). Instilled in the conjunctival sac of the eye was 50 µL of sample and the appearance of irritation was observed at the time of administration and after 1 h. The evaluation was undertaken by direct observation of the anterior segment of the eye calculating the injuries of the conjunctiva, iris and cornea. The punctuation applied has been described elsewhere [20] and the ocular irritation index has been calculated according to Equation (3).

(3) OII=Corneal (A×B×5)+Iris (A×5)+Conjunctiva (A+B+C)·2

### 2.7. In Vitro Customized Corneal Membrane Model and Interactions Study

#### 2.7.1. Langmuir Monolayers

Langmuir monolayers were obtained by spreading a lipid solution at the air/water interface. The subphase was a TRIS buffer solution (10 mM, pH 7.4). To prepare the spreading solution, appropriate amounts of ACMM (adult corneal membrane model) lipid mixture were dissolved in chloroform/methanol (2:1; final concentration 1 mM). ACMM composition was L-α-phosphatidylcholine 45%, L-α-phosphatidylethanolamine (55%), L-α-phosphatidylserine (10%); PC:PE:PS (45:55:10) at 50.4% mol/mol supplemented with sphingomyelin (25%) and cholesterol (75%) SM:CHOL (25:75) at 49.6% mol/mol. All lipids were from Avanti^®^Polar Lipids, (Alabaster, AL, USA). A Langmuir film balance (Biolin Scientific, Manchester, United Kingdom) equipped with a Whilhelmy filter plate and a Teflon barrier was used to obtain the surface-pressure (*π-A*) isotherms at the air water interface. The Teflon barrier (total area: 590 cm^2^) was placed on a vibration isolation table and enclosed in an environment protection cabinet. Chloroform/methanol lipid solution was spread dropwise on the subphase and before compression, 15 min were allowed in order to ensure total evaporation of the solvent. The reduction of the area was carried out by compression of monolayer at a constant rate of 10 cm^2^·min^−1^ at room temperature. Measurement accuracy was 0.1 mN·m^−1^.

#### 2.7.2. Preparation of Fluorescent Labeled LUVs

Fluorescent labeled large unilamellar vesicles (LUVs) were obtained by the extrusion method as described elsewhere [21]. Briefly, the lipid mixture used to produce ACMM was dissolved in a chloroform/methanol (2:1 *v*/*v*) mixture at 10 mM with a 0.1 mM of di-8-ANEPPS fluorescent probe (4-[2-[6-(dioctylamino)-naphthalenyl]ethenyl]-1-(3-sulphopropyl)-pyridinium) from Molecular Probes Inc. (Invitrogen, Cralsbad, CA, USA). The solvent was removed to dry in a vacuum with a rotary evaporator and then the dried film was kept at high vacuum overnight to eliminate any residual solvent traces. Dried film was rehydrated with a TRIS buffer solution (10 mM, pH 7.4) to obtain multilamellar vesicles (MLVs). Large unilamellar vesicles (LUVs) were prepared by 10 freeze–thaw cycles of MLVs, followed by the extrusion through polycarbonate filters (Nucleopore, Pleasanton, CA, USA) with 100 nm pore-size in a high-pressure system (Lipex Biomembranes, Vancouver, Canada). To assure LUVs formation, the temperature was maintained at 60 °C (above the transition temperature of lipids).

#### 2.7.3. Interaction of DXI NPs with Model Membranes

*π-A* isotherm experiments were carried out to ascertain the effect that NPs have when they make contact with the membrane model. Firstly, a lipid monolayer was obtained spreading 70 μL of 1 mM ACMM solution and after the equilibrium (at constant surface pressure, *π* = 0 mN·m^−1^) was reached at 35 μL of 1 mg/mL DXI NPs was spread on the lipid monolayer. The initial surface pressure increased by 20 mN·m^−1^, when equilibrium (constant surface pressure at 20 mN·m^−1^) was achieved. The monolayer compression continued until the collapse pressure (*π*_c_) was reached. There are some useful parameters to characterize the *π-A* curves; *π_c_* is the maximum surface pressure that can be achieved without breaking the film and the compressibility modulus ($C_{s}^{-1}$), which is a measure of the monolayer elasticity and characterizes the phase state of lipid films. It was calculated using Equation (4):

(4) $$C_{s}^{-1}=-A\left(\frac{\partial \pi }{\partial A}\right)$$

where *A* is the area occupied and *π* is the surface pressure. Values of $C_{s}^{-1}$ ranges from 12.5 to 50 mN m^−1^ for liquid-expanded phase and from 100 to 250 mN·m^−1^ aproximately for the liquid-condensed phase [22,23,24].

Dipole potential measures were performed by fluorescence spectroscopy. When NPs bind to penetrate the membrane, this causes changes in the membrane dipole potential, which can be determined by monitoring the fluorescence intensity excitation spectral shifts of the di-8-ANEPPS probe [25,26,27]. Fluorescence excitation spectra were recorded using Photon Technology International AC-10 spectrofluorometer (London, ON, Canada) at an emission wavelength was 580 nm. The measurement of the membrane dipole potential comes from the ratio of fluorescence intensities at *λ*_ex_460 nm and *λ*_ex_520 nm (*R = I_460_/I_520_*). For interaction assays, increasing amounts of NPs suspension (5–250 μg·mL^−1^) were added to 40 μL of di-8-ANEPPS labeled LUVs (200 μM lipid, 2 μM ANEPPS) and the variation of fluorescence intensity and fluorescence ratio (*R*) with NPs concentration were recorded. The normalized *R_norm_* was obtained by dividing the fluorescence ratio, *R*, obtained for a LUVs suspension in presence of NPs with that obtained in absence of them (*R_o_*). The data of *R_norm_* (*R/R_o_*) as a function of NPs concentration were fitted to a single binding site model Equation (5) using GraphPad Prism software (San Diego, CA, USA) [28]:

(5) $$1-R_{norm}=\frac{B_{max}\left[NPs\right]}{K_{d}\times [NPs]}$$

where *B_max_* is $\left(1-\frac{R_{min}}{R_{o}}\right)$*,* [NPs] is the NPs concentration (μg/mL) and *K_d_* is the apparent dissociation constant.

### 2.8. Prevention of In Vivo Ocular Inflammation

Prevention of ocular inflammation was assessed in vivo (*n* = 6/group) using New Zealand Albino rabbits. In order to carry out this assay, 50 µL of the developed formulations were applied to the conjunctival sac of the eye using the left eye as a control. After 30 min, 50 µL of sodium araquidonate 0.5% were applied. Inflammation was measured every 30 min and the ocular inflammation score (OII) was calculated as reported elsewhere [29].

### 2.9. Ex Vivo Ocular Permeation

In order to study the drug release from the NPs, Franz diffusion cells were used. Corneal and scleral tissue of male New Zealand rabbits (2.5–3 kg) was removed under veterinary supervision, and according to the Ethics Committee of Animals Experimentation from the University of Barcelona (CEEA-UB). The rabbits were anesthetized with intramuscular administration of ketamine HCl (35 mg/kg) and xylazine (5 mg/kg) and euthanized by an overdose of sodium pentobarbital (100 mg/kg) administered through marginal ear vein under deep anesthesia. The cornea and sclera were excised and immediately transported to the laboratory in artificial tear solution [30].

The tissues were placed in the cells with a separating membrane with a diffusional area of 0.64 cm^2^. The developed nanoparticles were placed on the donor compartment (0.2 mL DXI NPs) and the drug release from the basolateral compartment was studied at different timepoints by removing 300 µL of sample and replacing it with free PBS. The cumulative DXI amount permeated was calculated, at each time point, from the DXI amount in the receiving medium.

## 3. Results and Discussion

### 3.1. Characterization of the Nanocarriers

The formulations were characterized as seen in Table 1. The formulations were optimized using PLGA-PEG 5% for each surfactant used (Lutrol, PVA and Tween80^®^). Therefore, four different polymers and three of the most commonly used surfactants were assessed. Different PLGA-PEG triblocks were used.

As demonstrated in Table 1, all the formulations except F1 show an average size below 200 nm. In the case of F1, the average size was slightly bigger than the other formulations and the ZP was closer to neutrality. ZP was negative (around −15 mV) due to polymer carboxylic chain [10].

The same phenomenon was observed in all the formulations containing PVA, obtaining lower ZP than with the other surfactants used due to the fact that PVA would interact with PLGA chains causing an increase in ZP [31]. Regarding the EE, it was higher than 85% in all the formulations. This confirms that DXI was encapsulated in the polymeric matrix using all the polymers and surfactants.

### 3.2. DXI NPs Characterization Studies

**Figure 1:**
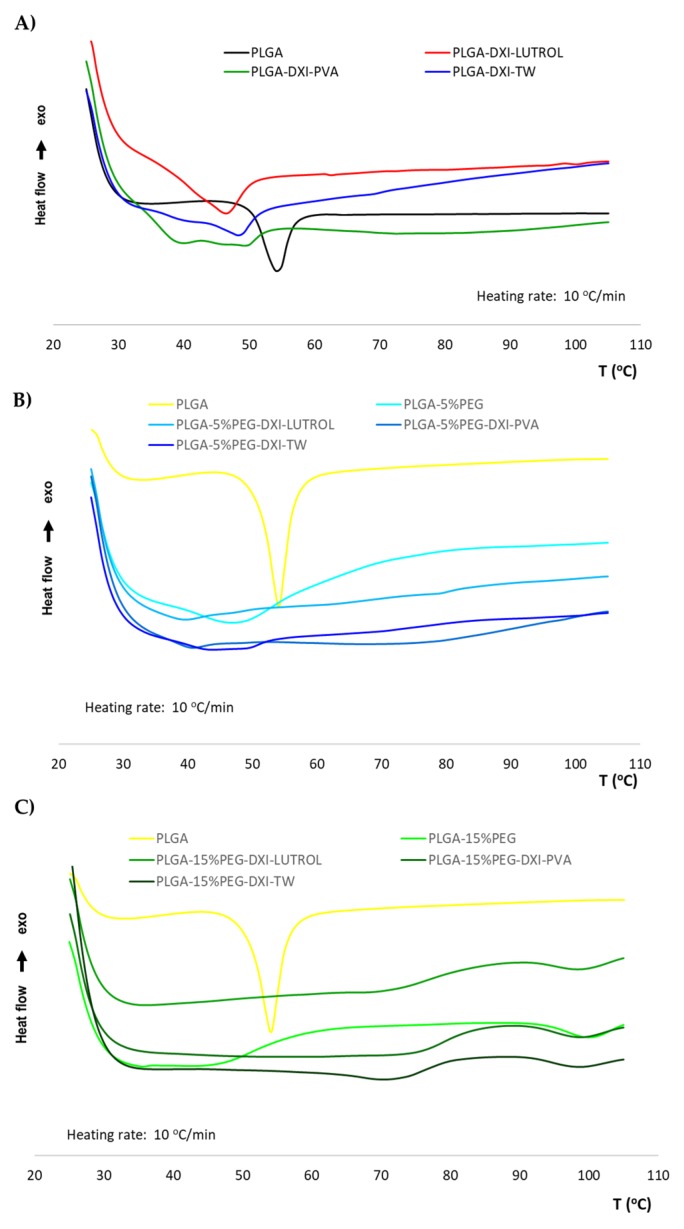
Differential scanning calorimetry (DSC) of DXI loaded nanoparticles. (**A**) DXI PLGA nanoparticles (NPs), (**B**) DXI PLGA 5% PEG NPs and (**C**) DXI PLGA 15% NPs.

DSC profiles of DXI show a sharp endotherm corresponding to its melting transition (data previously published), characterized by a *T*_max_ = 53.06 °C, which was not detected in DXI-PLGA-PEG NPs [11]. This suggests that DXI formulated in PLGA or in PLGA-PEG NPs is in an amorphous or disordered crystalline phase of a molecular dispersion or in a solid solution state in the polymer matrix (Figure 1). Moreover, PEG masks PLGA glass transition temperature (*T*_g_) probably due to the plasticizing effect of PEG. This effect has also been shown to cause a reduction in the attractive forces amongst the polymer chains leading to slight reduction in the *T*_g_ value of the polymer, which can be observed on the thermic profile [32].

**Figure 2:**
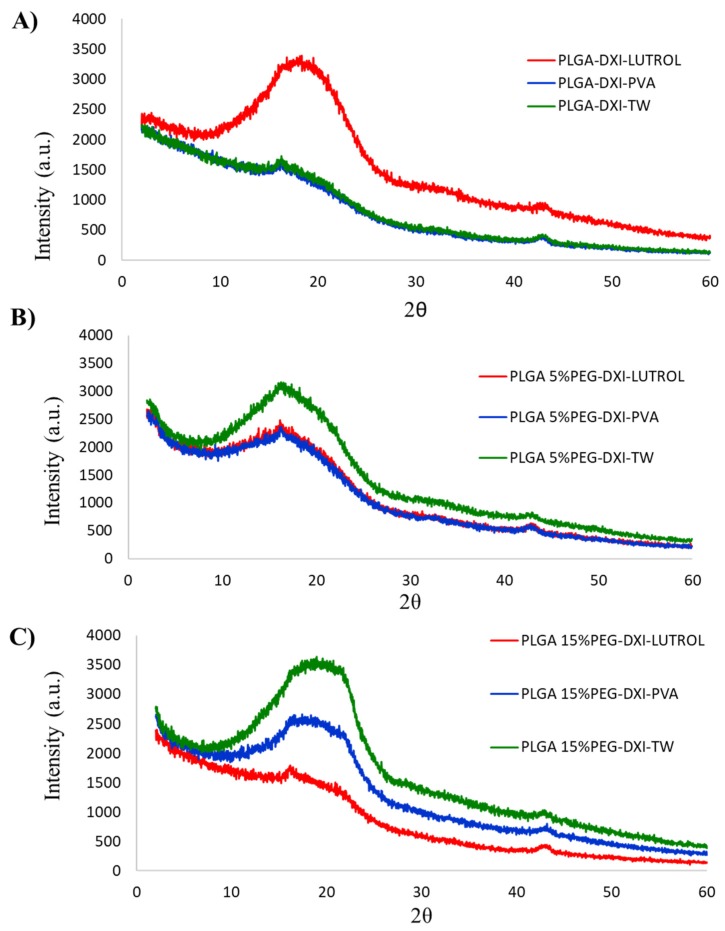
X-ray diffraction of DXI loaded nanoparticles. (**A**) DXI PLGA NPs, (**B**) DXI PLGA 5% PEG NPs and (**C**) DXI PLGA 15% PEG NPs.

XRD profile (Figure 2) shows that all nanoparticles possess a similar profile due to the PLGA polymer, corresponding to an amorphous pattern and a peak at approximately 2θ = 18 °C is shown.

Moreover, no peaks corresponding with DXI are observed in any of the formulations. This confirms DXI encapsulation into the polymeric matrix [20]. Surprisingly, in DXI PLGA Lutrol NPs, the intensity obtained was higher than with PVA and Tween nanoparticles. The opposite phenomenon was found when adding PEG and with PLGA 5% PEG NPs; Tween shows an increased intensity against the other two surfactants. Furthermore, PLGA 15% PEG nanoparticles show a similar trend to PLGA 5% but with a markedly increased intensity of PLGA 15% Tween NPs. In this case, PVA NPs show an increased intensity than Lutrol. These results may indicate that in PLGA Lutrol NPs, the surfactant favored an increased order of the matrix whereas in PEG NPs, Tween increases the order, obtaining higher intensities [33]. This interaction of Tween and PEG was confirmed as the percentage of PEG increased.

**Figure 3:**
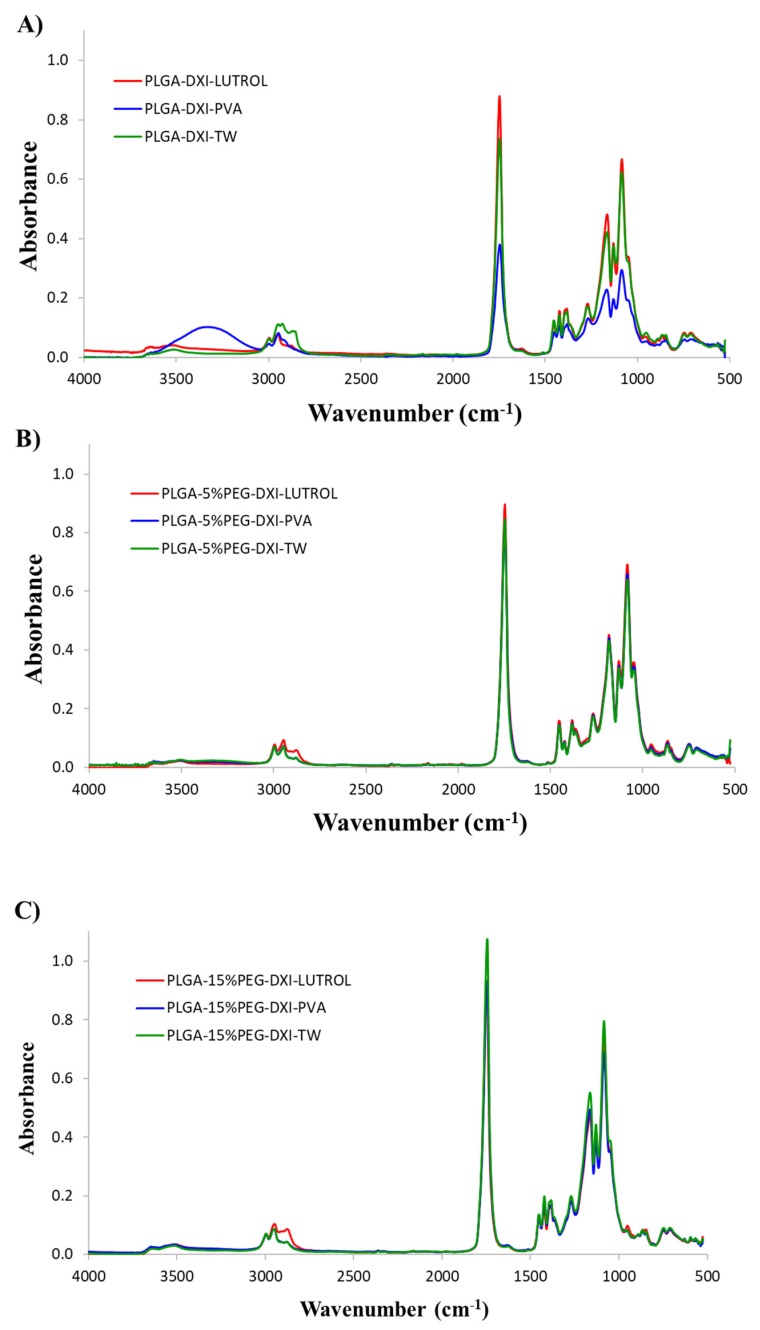
FTIR of DXI loaded nanoparticles. (**A**) DXI PLGA NPs, (**B**) DXI PLGA 5% PEG NPs and (**C**) DXI PLGA 15% NPs.

FTIR analysis was used to study the interactions between the drug, the surfactant and polymer (Figure 3). There was no evidence of strong bonds between DXI and PLGA-PEG and the surfactant. DXI peaks (reported in previous publications) were not found in the NPs. These results meaning that DXI was encapsulated in the polymeric matrix. Moreover, in the case of PLGA NPs using PVA, a peak around 3400 cm^−1^ corresponding with PVA can be observed. This may be due to a slight amount of PVA on the NPs surface due to the steric interaction between PVA and PLGA. PEG is probably able to reduce these interactions and no PVA peaks were found in the formulations using PEG. Furthermore, PLGA exhibited intense bands at 2907 and 2950 cm^−1^. An intense peak at 1743 cm^−1^ is shown in all DXI NPs samples, thus corresponding to the C-O stretching vibration of the carbonyl groups present in the two monomers that form the PLGA matrix [9]. Bands obtained 1077, 1199 and 1305 cm^-−1^ in the NPs are attributed to stretching vibrations of the OH group of the polymer [9].

**Figure 4:**
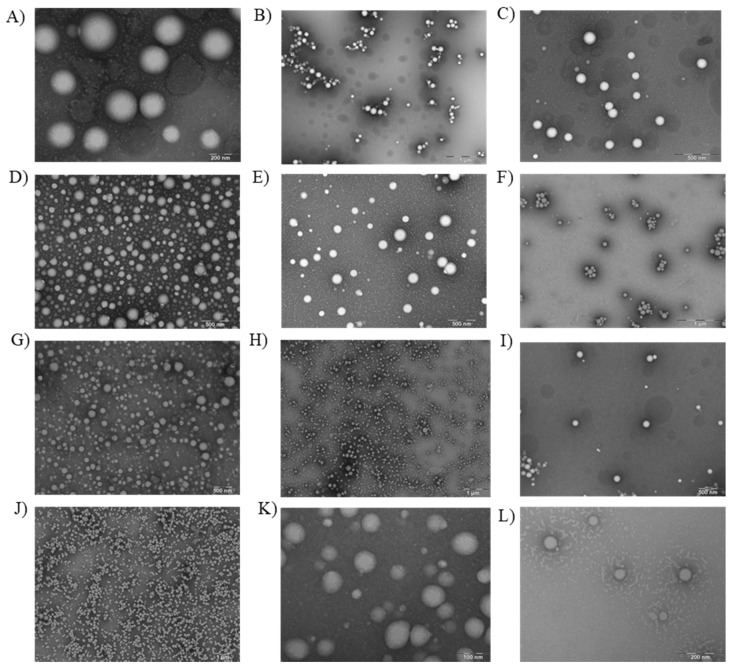
Transmission electron microscopy of the different formulations of nanoparticles (**A**) DXI PLGA NPs using Lutrol; (**B**) DXI PLGA NPs using PVA; (**C**) DXI PLGA NPs using Tween80^®^; (**D**) DXI PLGA5%PEG NPs using Lutrol; (**E**) DXI PLGA 5% PEG NPs using PVA; (**F**) DXI PLGA 5% PEG NPs using Tween80^®^; (**G**) DXI PLGA 10%PEG NPs using Lutrol; (**H**) DXI PLGA 10% PEG NPs using PV; (**I**) DXI PLGA 10% PEG NPs using Tween80^®^; (**J**) DXI PLGA15%PEG NPs using Lutrol; (**K**) DXI PLGA 15% PEG NPs using PVA and (**L**) DXI PLGA 15% PEG NPs using Tween80^®^ as a surfactant.

In addition, the formulations of DXI loaded NPs were observed using TEM [34]. As can be observed in Figure 4, all the formulations show a spherical shape and a smooth surface. No differences regarding the PEG chains or between the surfactants were observed. In addition, there was no sign of aggregation phenomena and the average size was similar to that obtained using photon correlation spectroscopy.

### 3.3. Sterilization Using Gamma Radiation

PLGA nanoparticles can be effectively sterilized for ocular drug delivery using gamma radiation [35]. However, in some cases, gamma radiation can affect physicochemical parameters of the formulations. For this reason, the formulations were analyzed before and after gamma radiation and the ratio between both was calculated. As shown in Table 2, the majority of the formulations were not affected by the irradiation and only PI was slightly increased in F1, which was the only formulation with more than 200 nm of average size. However, the ratio before and after was close to 1 in all cases. These results were in agreement with other authors that freeze dried PLGA NPs such as Ramos et al. confirming that 25 KGy does not affect the physicochemical structure of DXI NPs [36].

### 3.4. In Vitro Drug Release

The in vitro release profile of DXI from the NPs demonstrates that all the formulations have a kinetic profile that is characteristic of prolonged drug release formulations (Table 3) [37]. As expected, DXI free is released after 150 min. The release best fit was a one-phase association, meaning that the release is proportional to the DXI concentration with a half-life of 29 min [38]. This fast release implies that several administrations would be necessary in order to prevent and treat the inflammation associated with several pathologies and associated with surgical procedures.

**Figure 5:**
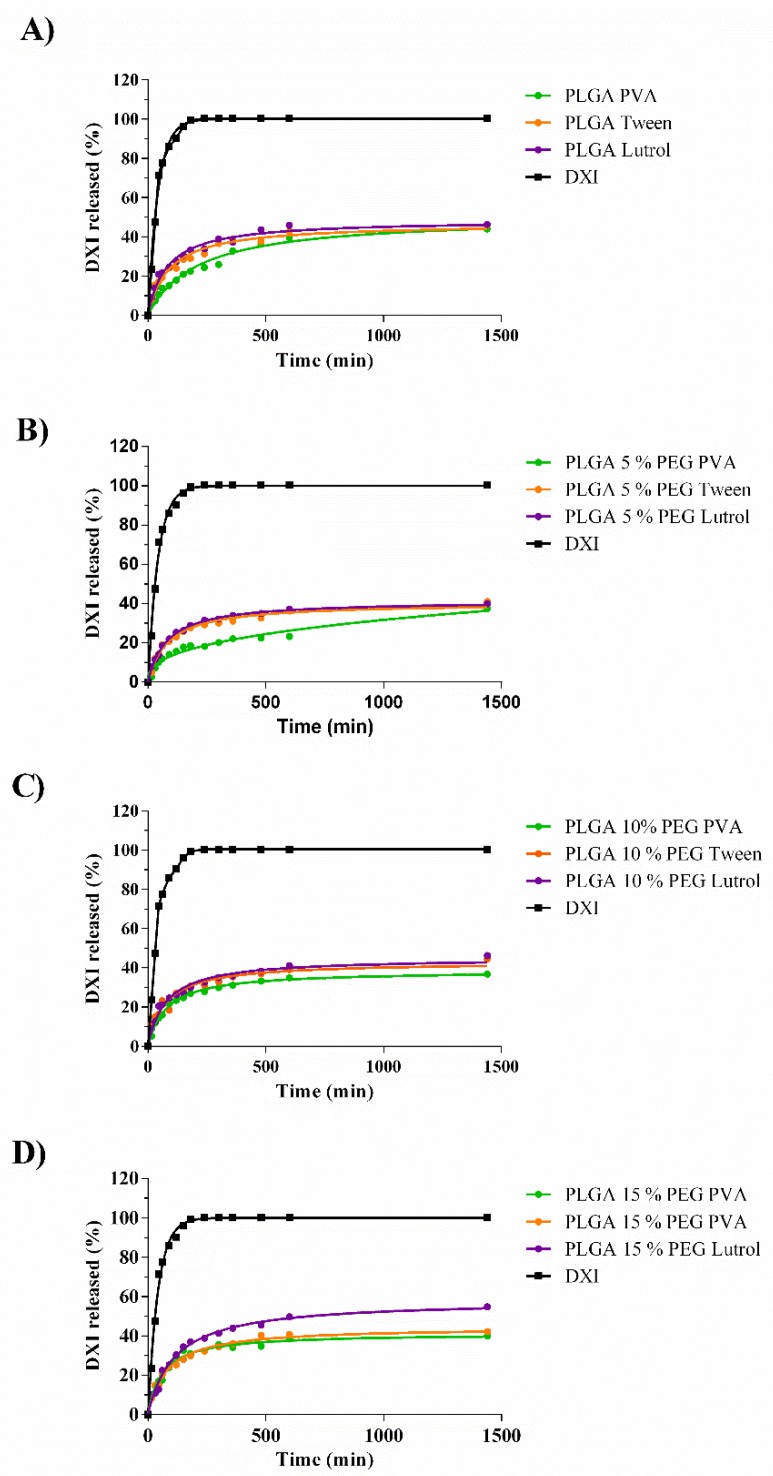
In vitro drug release of PLGA NPs using different PEG % and surfactants. (**A**) DXI PLGA NPs, (**B**) DXI PLGA 5% PEG NPs, (**C**) DXI PLGA 10% NPs and (**D**) DXI PLGA 15% NPs.

In all the DXI NPs, it can be observed that the profile release is slower, and two phases can be distinguished (Figure 5). The initial rapid release lasts around 150 min in all the formulations, associated with the drug attached to the NPs surface. After 150 min it was observed that NPs solutions maintained a sustained drug release profile and were able to release the drug for more than 24 h (1440 min). The drug release (Y in percentage) from the formulations was adjusted to a hyperbola equation, Equation (6)

(6) $$Y=\frac{B_{\max}\times t}{\left(K_{d}+t\right)}$$

Being the K_d_ half of the time when DXI that is released at equilibrium and B_max_ the maximum % of DXI released after 24 h of experiment.

According to the results showed in Table 3, PLGA nanoparticles and PLGA 5% NPs show that the dissociation constant (K_d_) decreased obtaining higher K_d_ from PVA > Tween80 > Lutrol F68. These results indicate that DXI PLGA PVA NPs had a slower release when compared to the other two surfactants [39]. The same trend was observed with PLGA 5% PEG. However, it was reversed as the % of PEG increases and with 10% PEG all the K_d_ obtained with the different surfactants were similar. In the formulations containing 15% PEG, the initial trend was reversed obtaining K_d_ of Lutrol F68 < Tween80 < PVA. Therefore, as increasing the PEG concentration, PVA might probably decrease the steric interactions with PLGA and Lutrol NPs release DXI slowly. Moreover, comparing the polymers used, increasing the amount of PEG increased the value of B_max_ irrespective of the surfactant used. In this sense, the lower B_max_ corresponded to PVA DXI loaded PLGA 15% PEG NPs.

A sustained release of DXI into the NPs was shown in all the formulations compared with the free DXI and it would decrease the number of drug administration required by a patient.

### 3.5. Ocular Tolerance

**Figure 6:**
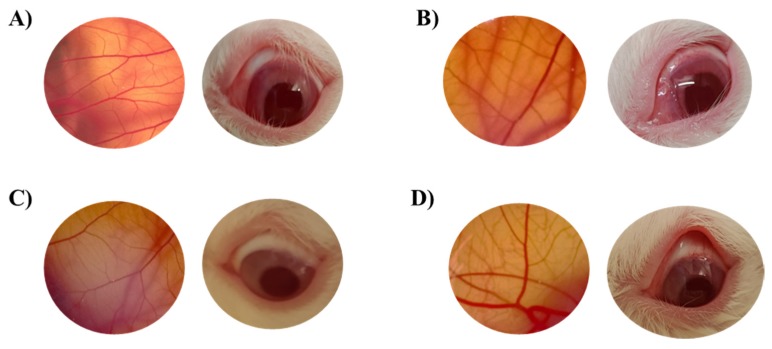
In vitro and in vivo irritation assay. (**A**) Lutrol DXI PLGA NPs, (**B**) Lutrol DXI PLGA 5% PEG NPs, (**C**) Lutrol DXI PLGA 10% PEG NPs and (**D**) Lutrol DXI PLGA 15% PEG NPs.

Ocular tolerance was studied in vitro and in vivo. Hen’s egg-chorioallantoic membrane (HET-CAM) test was applied showing that the positive controls (NaOH 1 M) resulted in severe hemorrhage, which increased over five minutes grading this solution as a severe irritant. On the other hand, application of the formulations to the chorioallantoic membrane did not cause irritation and therefore, the formulations were classified as non-irritant. However, since a single in vitro test is not able to reproduce the in vivo situation, ocular tolerance Draize tests were performed. The tests were carried out with all DXI NPs. As an example, in Figure 6, DXI NPs containing Lutrol are shown.

In this sense, none of the developed formulations were irritant in vivo or in vitro. These results correlate with previous studies developed using PLGA NPs [40]. These results are in agreement with the HET-CAM test confirming the non-irritant potential of DXI loaded polymeric nanoparticles and also the suitability of the HET-CAM test in order to mimic the in vivo irritation phenomena.

### 3.6. In Vitro Interactions with a Customized Corneal Membrane Model

#### 3.6.1. π-A Isotherm Analysis

**Figure 7:**
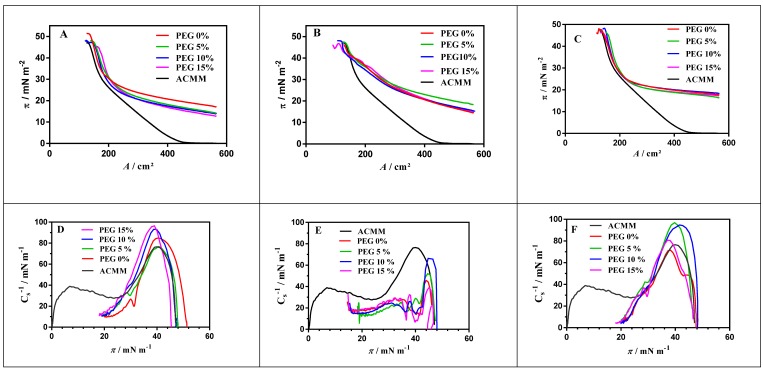
Surface pressure-area (*π*-A) isotherms of the ACMM monolayer and mixed ACMM–NPs_DXI_PLGA at different PEG concentrations and surfactants (**A**) PVA, (**B**) Tween and (**C**) Lutrol, (**D**–**F**) respective compressibility modulus plots.

Figure 7 shows the *π-A* curve obtained for lipid monolayer (ACMM composition) and the curves obtained when DXI NPs were spread on the ACMM-lipid monolayer. In this case, the initial surface pressure was increased until equilibrium (constant surface pressure) was reached. The posterior compression of the system showed the changes in the area until collapse pressure was achieved (Figure 7). DXI NPs caused an expansion of the ACMM monolayer in all the cases being reflected by a pressure shifting to the right side of the graph. The area increased depending on the surfactant used (*A*_TW_ > *A*_PVA_ > *A*_LUT_) as can be seen in Table 4. It shows area values at 32 mN·m^−1^, which is accepted for biological membrane and bilayers pressure [41,42]. This area expansion suggests that the NPs interact with the membranes by inserting themselves between the lipids. DXI NPs developed using Tween as a surfactant present greater area expansion, obtaining 234.8 cm^2^ at 32 mN·m^−1^ (significantly greater than the 169.5 cm^2^ obtained for ACMM). PLGA-PVA NPs and PLGA-Lutrol NPs, at the same surface pressure, have 185.5 cm^2^ and 178.2 cm^2^ respectively suggesting a more subtle expansion than the former. Regarding the percentage of PEG in the formulations, this affects the *A* values but not significantly. However, to deeply understand this data, the compression modulus was calculated using Equation (4).

ACMM monolayer exhibits a LE (liquid expanded) state in the entire range of surface pressures (Figure 7). $C_{s}^{-1}$ of ACMM monolayer constantly grew until a first maximum (π around 14 mN·m^−1^) then decreased slowly (plateau between 15 and 25 mN·m^−1^). Afterwards, it started rising again until a second maximum (π around 47 mN·m^−1^), finally $C_{s}^{-1}$ decreasing drastically because of the monolayer collapse. This behavior has been observed for other lipid systems containing cholesterol [39,40] and it is indicative of the changes in the packing of the monolayer during compression and clearly indicates two membrane domain formation, a phospholipid-rich domain (low $C_{s}^{-1}$ values) and a cholesterol-rich domain (high $C_{s}^{-1}$ values). This pattern is similar to other systems studied; in all cases two LE domains appeared. Collapse pressure values are very similar and do not differ significantly from the *π*_c_ obtained for pure cholesterol monolayer (around 46 mN·m^−1^) [43]. This suggests some immiscibility phenomena [43,44,45] according with the presence of two domains. $C_{s}^{-1}$ values allow us to postulate that PEG mainly mixes with phospholipid-rich domains. However, PEG modifications do not seem to affect in a significant manner. Despite this, a trend towards a higher interaction as PEG increased could be observed. On the other hand, Tween 80 clearly expanded the monolayer (more than Lutrol and PVA).

#### 3.6.2. Membrane Dipole Potential Changes

**Figure 8:**
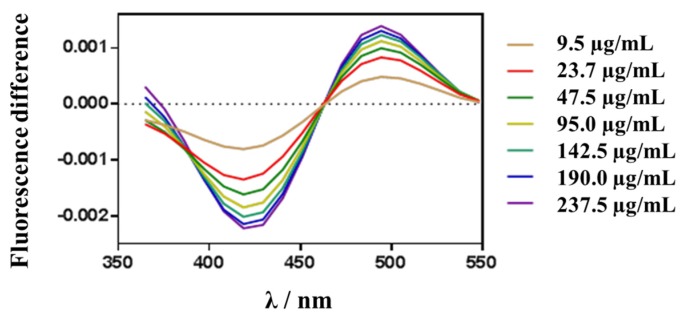
Fluorescence differential spectra of di-8ANEPPS labeled ACMM large unilamellar vesicles (LUVs) in presence of DXI NPs using Tween, at different concentrations. Before subtraction, the spectra were normalized to the integrated areas. Lipid concentration was constant at 200 µM. All experiments were performed in 10 mM TRIS buffer (pH 7.4) at 32 °C.

**Figure 9:**
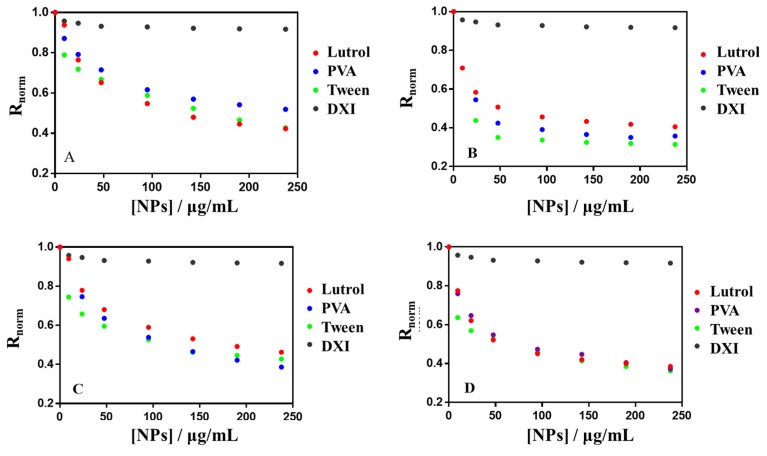
Variation of di-8-ANEPPS excitation normalized ratio (*R/R_o_*) as a function of the surfactant used and PEG content in DXI NPs. (**A**) Without PEG, (**B**) 5% PEG, (**C**) 10% PEEG and (**D**) 15% PG. Lipid concentration was 400 μM. Black dots correspond to DXI effect on *R_norm._* Experiments were performed at 32 °C.

The dipole potential of lipid membranes is an electrical potential caused by the dipole orientation at the membrane/water interface. As described in the literature, polar groups of phospholipid molecules, such as ester, bond with acyl chains and glycerol groups of phospholipids. The orientation of water molecules around the membrane is the origin of the membrane dipole potential [46]. This is a positive potential in the bilayer and plays an important role in membrane functions, affecting the membrane permeability and the drugs binding capacity to the membrane among others [28,47,48]. Fluorescent probe di-8-ANEPPS is sensitive to the membrane dipole potential changes. It was used to investigate the interaction of NPs with ACMM. Regarding the NPs composition, it is possible to evaluate the effect of surfactant and the percentage of PEG in dipole potential changes. DXI NPs were tested at 32 °C and all of them were able to reduce the membrane dipole potential. A decreasing dipole potential suggests a shift to the red in the excitation spectra that results in a differential fluorescence spectrum, obtained by subtracting the ACMM-liposome spectrum from that obtained for a suspension containing ACMM-liposomes and increasing concentrations of DXI NPs. The extension of dipole potential reduction is concentration dependent. Figure 8 shows the difference fluorescence spectra obtained for a suspension containing ACMM-liposomes and increasing concentrations of DXI NPs developed using Tween. The interaction of all other formulations assessed causes similar redshift in the excitation spectra yielding a similar shape in differential fluorescence spectra. Figure 9 shows the binding profile obtained by plotting the di-8-ANEPPS excitation normalized ratio (*R_norm_*) NPs concentration (in μg·mL^−1^). *R_norm_* slightly decreased with increasing of the NPs concentration implying a drop off on membrane lipid order. Due to the fact that from 95 µg·mL^−1^ the interaction of DXI NPs and the in vitro membrane is close to saturation; the extrapolation of this results in vivo, would ensure that the concentration added in the eye would cause the maximum interaction with the corneal membrane, ensuring the maximum interaction of DXI NPs with the membrane. This is agreement with *π-A* results being both models in accordance. Table 5 shows the interaction parameters (*K_d_* and *B_max_*) for all formulations assayed. *K_d_* is an index of the interaction, the lower *K_d_* the greater interaction, and *B_max_* indicates the magnitude of binding maximum. Regarding the surfactant used, the highest interaction is associated with Tween in all the cases.

### 3.7. Ocular Inflammation

Ocular inflammation is a well-documented complication of cataract surgery, causing increased intraocular pressure, posterior-capsule opacification, cystoid macular edema and decreased visual acuity [49]. Therefore, prevention of ocular inflammation has been studied in vivo using New Zealand rabbits. 

**Figure 10:**
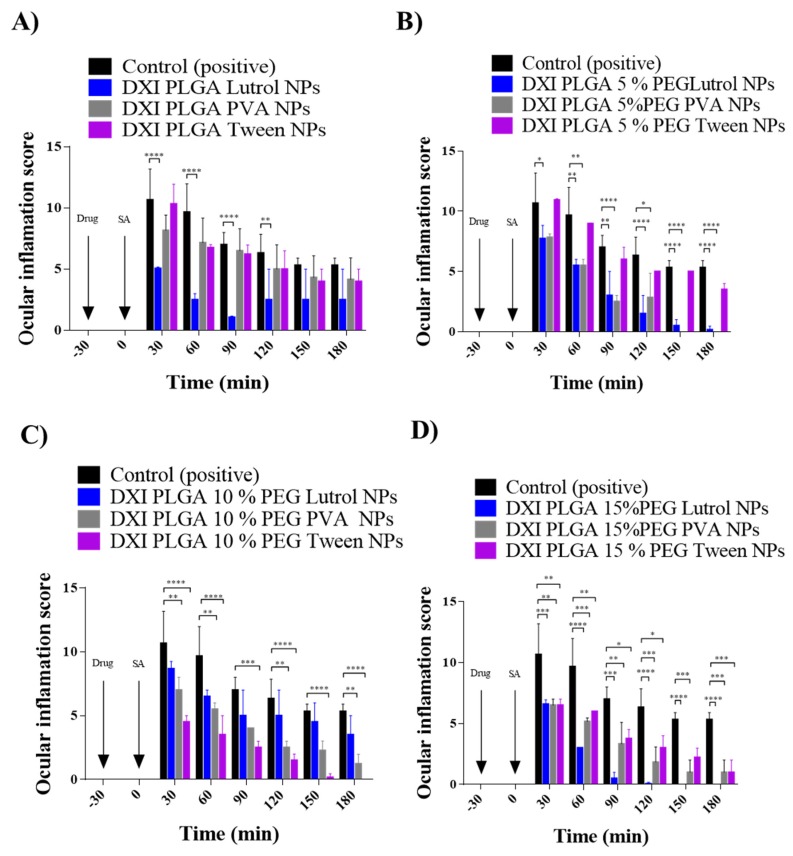
Inflammation score obtained with the different formulations. (**A**) DXI PLGA NPs, (**B**) DXI PLGA 5% PEG NPs, (**C**) DXI PLGA 10% PEG NPs and (**D**) DXI PLGA 15% PEG NPs (* *p* < 0.05, ** *p* < 0.01, *** *p* < 0.001; **** *p* < 0.0001) significantly different antiinflammatory effect).

It can be observed that adding PEG to the formulations increases their therapeutic efficacy probably due to the fact that it increases the penetration of the nanoparticles into the corneal epithelium by interpenetration and/or hydrogen bonding with the mucus [5]. Moreover, DXI NPs were able to prevent ocular inflammation more effectively that free DXI in all the formulations [11]. In the case of DXI NPs prepared with PVA, a difference could be observed when PEG was added but no difference was observed as the percentage of PEG increased. In the case of PLGA NPs prepared using Tween, their efficacy increased with PEG 10% and no difference between PEG 10% and 15% was appreciated. On the other hand, NPs prepared using Lutrol showed the best results when either high amount of PEG or even no PEG was applied. These results correlated with the in vitro drug release where PLGA Lutrol NPs were able to obtain higher release values (Figure 10). These therapeutic efficacy studies demonstrated that when the anti-inflammatory efficacy was compared using PEG, this PEG addition was able to increase the antiinflammatory effects. Therefore, the maximum therapeutic effect was obtained with formulations containing PEG compared with only PLGA. Moreover, the necessary amount of PEG on the surface of the NPs for optimal permeability in the mucosa will vary depending on different factors such as size, NPs core (in this case PLGA), the media in which NPs are dispersed, the molecular weight of PEG and the type of mucosa with which they interact [50]. Therefore, in our study, the differences observed using several PEG percentages obtaining greater antiinflammatory efficacy might be due to the presence of different surfactants. 

The drug release of PLGA 10% NPs using Tween shows similar values, with Tween and Lutrol having a slightly better Lutrol drug release. However, the in vivo results demonstrated that PLGA 10% Tween decreased the inflammation more effectively than PLGA 10% Lutrol. This correlated with the in vitro interactions study since it shows that Tween increased membrane permeability and might lead to the obtention of increasing antiinflammatory effects. Regarding PLGA 15%, higher amounts of DXI in vitro were also released and the best antiinflammatory results were observed with this formulation.

Therefore, the best anti-inflammatory results were obtained with PLGA Lutrol, PLGA 5% PEG Lutrol, PLGA 10% PEG Tween and PLGA 15% PEG using Lutrol. 

In this sense, the use of Lutrol F68 as a surfactant is supported by a high number of authors for ocular drug delivery. This may be due to the fact that it favors both the physical properties of the NPs and, at the same time, it presents low ocular toxicology as has been demonstrated, due to the absence of any irritating ocular phenomena even in high concentrations [51]. Moreover, this surfactant is able to increase the anti-inflammatory efficacy of the NSAIDs formulation [52].

### 3.8. Ex Vivo Permeation Assay

**Figure 11:**
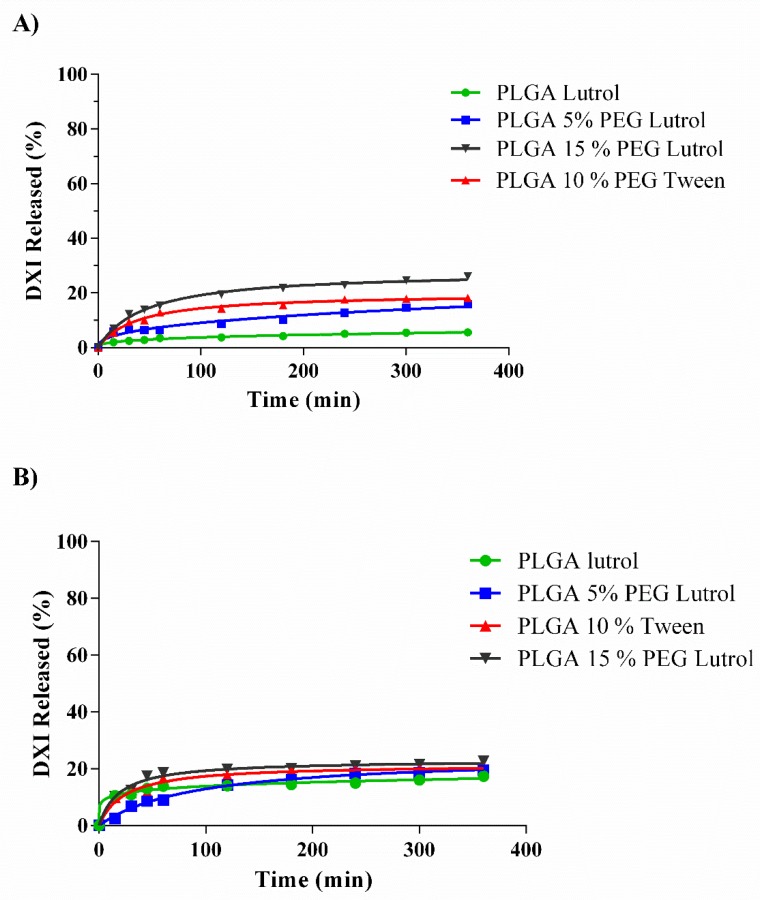
Ex vivo permeation assay of the most effective DXI loaded nanoparticles using different surfactants (PLGA Lutrol, PLGA 5% PEG Lutrol, PLGA 10% PEG Tween and PLGA 15% PEG using Lutrol). (**A**) Corneal permeation; (**B**) Scleral permeation.

To reduce the number of animals, an ex vivo permeation assay was carried out using the most effective formulations. Therefore, four formulations were assessed (DXI PLGA Lutrol NPs, DXI PLGA 5% PEG Lutrol NPs, DXI PLGA 10% Tween NPs and DXI PLGA 15% PEG Lutrol NPs). Corneal and scleral permeation were studied (Table 6, Figure 11). All the release data were adjusted to the most common kinetic equations.

In all formulations assessed, corneal DXI permeation was slightly higher than scleral permeation. These results were in accordance with other authors developing polymeric NPs containing NSAIDs, which were able to promote increased corneal retention [53]. Focusing on this corneal permeation, the best fit was obtained for both PLGA and PLGA 5% PEG NPs with a Korsmeyer–Peppas equation. In this equation, the release exponent values (n) are higher with PLGA 5% PEG and also the K was higher on PLGA 5% (0.81 vs. 1.45 min). These results suggest that the addition of 5% PEG increases corneal DXI release. At the same time, it was demonstrated using the customized corneal membrane model that the addition of PEG slightly increased the interactions. Increasing the PEG % to 10% and 15%, adjusted better with a hyperbola equation. Increasing PEG % also increased the amount of DXI permeated. Moreover, it also increased the K_d_, obtaining a slower release as PEG % is increased. These results were in concordance with the in vitro drug release, where a higher amount of DXI was released but also a longer time until it reached the equilibrium was observed.

Moreover, the results obtained correlate with other studies such as the investigation carried out by Giannavola and colleagues where they demonstrated that PEGylation of polymeric NPs using PLA as the core polymer, increased drug permeation through the corneal tissue [54]. Moreover, they obtained higher drug values in aqueous humor with PEGylated NPs than with NPs without PEG.

Regarding the scleral permeation, PLGA NPs were able to permeate more efficiently through the cornea than through the sclera showing a certain corneal tropism. On the other hand, PLGA 5% PEG permeated slightly better through the scleral tissue. Adding more PEG marginally increased scleral permeation but significantly increased corneal values. These results were in agreement with the results obtained using the customized lipid model. All the formulations with PEG were adjusted to a Hyperbola equation, obtaining lower K_d_ as the PEG % increased (faster release of the drug). 

Therefore, it is worth noting by observing the results of ex vivo permeation studies that nanoparticles with the presence of PEG have great potential to increase ocular bioavailability in the anterior segment of the eye, such as the cornea, conjunctiva or aqueous humor. Therefore, the use of PEGylated NPs will require a lower dosing frequency, allowing the mucin to retain a higher concentration of DXI. This may indicate that the patients will follow a better compliance with the treatment, which will be translated into greater therapeutic efficacy [55].

## 4. Conclusions

DXI, the active enantiomer of ibuprofen, encapsulated in biodegradable PLGA nanoparticles, constituted a suitable strategy to prevent corneal inflammation, associated with procedures such as cataract surgery. Interaction studies of DXI NPs using different surfactants and PEG amounts were carried out using a customized in vitro membrane model. Using this model, it was observed that DXI NPs were able to interact with the membrane in an effective manner in all cases. However, Tween 80 could expand the membrane by potentially increasing its permeability, which could be useful for drug delivery to the posterior eye segment. Moreover, a comparison between different surfactants shows that Lutrol produced the best therapeutic efficacy, which may be due to a synergistic, anti-inflammatory effect being elicited [56]. It has also shown a favorable interaction with the developed membrane model. Moreover, increasing the amount of PEG constitutes an effective strategy to increase corneal drug delivery without causing irritation of the ocular tissues.

In conclusion, the formulations containing DXI PLGA 15% PEG prepared using Lutrol as a surfactant were able to release DXI more effectively for the treatment of ocular inflammation. The development of an in vitro membrane model constitutes an interesting strategy in order to correlate in vitro, ex vivo and in vivo results and, in further experiments, reduce the number of animals required.

## Figures and Tables

**Table 1:** Characterization of the different formulations developed.

Formulation Number	Polymer Used	Surfactant	Average Size (nm)	Polydispersity Index (PI)	Zeta Potential (ZP, mV)	EE (%)
1	PLGA 503 H	PVA	234.1 ± 0.5	0.081 ± 0.009	−12.2 ± 1.3	93.4
2		Tween80^®^	146.0 ± 0.6	0.054 ± 0.008	−25.2 ± 0.6	87.5
3		Lutrol	159.5 ± 0.8	0.058 ± 0.021	−26.0 ± 0.1	85.1
4	PLGA-5%	PVA	167.1 ± 1.1	0.080 ± 0.012	−11.8 ± 0.9	95.0
5		Tween80^®^	138.4 ± 1.3	0.072 ± 0.015	−14.1 ± 1.1	91.5
6		Lutrol	154.2 ± 1.9	0.063 ± 0.015	−18.7 ± 1.4	93.8
7	PLGA 10%	PVA	140.9 ± 1.0	0.055 ± 0.023	−16.7 ± 0.7	99.0
8		Tween80^®^	119.2 ± 1.0	0.074 ± 0.008	−21.2 ± 0.6	99.2
9		Lutrol	120.7 ± 0.8	0.071 ± 0.008	−23.1 ± 1.8	91.5
10	PLGA 15%	PVA	156.4 ± 0.8	0.078 ± 0.008	−16.2 ± 0.7	92.2
11		Tween80^®^	143.0 ± 0.5	0.062 ± 0.006	−21.4 ± 0.8	93.4
12		Lutrol	155.2 ± 1.1	0.076 ± 0.012	−22.5 ± 0.5	94.0

**Table 2:** Sterilization using gamma radiation (ratio between before and after sterilization).

Formulation Number	Polymer Used	Surfactant	Average Size Ratio	Polydispersity Index Ratio	Zeta Potential Ratio	EE Ratio
1	PLGA 503 H	PVA	1.00	0.61	0.97	1.00
2		Tween80^®^	1.01	1.23	0.97	1.01
3		Lutrol	1.00	1.00	1.00	1.02
4	PLGA-5%	PVA	1.01	1.03	1.14	0.98
5		Tween80^®^	1.00	0.92	1.05	1.01
6		Lutrol	1.01	1.07	0.97	0.95
7	PLGA 10%	PVA	0.98	1.00	1.03	0.98
8		Tween80^®^	0.99	0.94	1.01	0.98
9		Lutrol	0.99	1.21	0.99	0.99
10	PLGA 15%	PVA	0.99	1.28	1.02	1.04
11		Tween80^®^	1.02	1.06	1.03	1.01
12		Lutrol	1.00	1.01	0.94	1.02

**Table 3:** Pharmacokinetic parameters of PLGA nanoparticles applied to a hyperbola equation.

Formulation Number	Polymer Used	Surfactant	B_max_ (%)	K_d_ (min)
1	PLGA 503 H	PVA	50.6 ± 2.3	210.6 ± 24.3
2		Tween80^®^	46.9 ± 1.6	89.6 ± 10.1
3		Lutrol	48.7 ± 1.4	79.7 ± 7.9
4	PLGA-5%	PVA	33.5 ± 2.8	149.2 ± 35.2
5		Tween80^®^	40.4 ± 1.1	84.5 ± 7.6
6		Lutrol	41.6 ± 0.5	79.9 ± 3.4
7	PLGA 10%	PVA	38.5 ± 0.5	78.1 ± 3.9
8		Tween80^®^	43.0 ± 1.8	72.1 ± 10.9
9		Lutrol	45.1 ± 1.4	77.1 ± 8.5
10	PLGA 15%	PVA	41.4 ± 1.1	59.8 ± 6.0
11		Tween80^®^	44.6 ± 1.0	79.4 ± 6.3
12		Lutrol	58.3 ± 1.5	113.6 ± 9.2

**Table 4:** Surface properties of ACMM only and mixed ACMM–DXI NPs monolayers containing different PEG concentrations; and PVA, Tween and Lutrol as surfactants.

Monolayer Composition	*A*/cm^2^ at 32 mN·m^−1^	$C_{s}^{-1}$/mN·m^−1^ at 32 mN m^−1^	*π_c_*/mN·m^−1^	$C_{s,\;max}^{-1}$/mN·m^−1^
ACMM	169.47	44.87	47.07	76.57
ACMM + PLGA-DXI-PVA-NPs	185.50	29.35	51.00	84.83
ACMM + PLGA-DXI-PEG 5%-PVA-NPs	194.17	39.89	47.72	76.77
ACMM + PLGA-DXI-PEG 10%-PVA-NPs	184.36	50.19	47.14	93.17
ACMM + PLGA-DXI-PEG 15%-PVA-NPs	197.36	60.17	45.45	96.46
ACMM + PLGA-DXI-TWEEN-NPs	234.10	28.16	46.98	45.88
ACMM + PLGA-DXI-PEG 5%-Tween-NPs	238.04	21.58	47.55	52.07
ACMM + PLGA-DXI-PEG 10%- Tween -NPs	223.49	23.52	48.02	66.36
ACMM + PLGA-DXI-PEG 15%- Tween -NPs	247.55	29.16	46.24	39.12
ACMM + PLGA-DXI-Lutrol-NPs	178.19	52.24	46.00	63.94
ACMM + PLGA-DXI-PEG 5%- Lutrol -NPs	186.69	47.55	47.30	96.83
ACMM + PLGA-DXI-PEG 10%- Lutrol-NPs	178.75	48.04	47.70	94.78
ACMM + PLGA-DXI-PEG 15%- Lutrol -NPs	180.36	40.82	46.56	63.94

**Table 5:** Binding parameters of PLGA DXI NPs using the fluorescence of the potential sensitive probe di-8-ANEPPS. *K_d_* is the apparent dissociation constant for binding NPs to ACMM and *B_max_* the maximum NPs capacity to link to the membrane.

DXI NPs	*B_max_*	*K_d_*	*R^2^*
PLGA-PVA	0.55 ± 0.01	39.95 ± 3.58	0.996
PEG 5%-PVA	0.68 ± 0.01	10.93 ± 1.07	0.998
PEG 10%-PVA	0.72 ± 0.02	46.98 ± 3.85	0.997
PEG 15%-PVA	0.65 ± 0.02	18.97 ± 2.03	0.993
PLGA-Tween	0.59 ± 0.00	27.52 ± 7.37	0.960
PEG 5%-Tween	0.70 ± 0.01	5.48 ± 0.62	0.999
PEG 10%-Tween	0.59 ± 0.02	16.17 ± 2.69	0.984
PEG 15%-Tween	0.62 ± 0.02	8.86 ± 1.95	0.976
PLGA-Lutrol	0.71 ± 0.03	57.49 ± 7.08	0.993
PEG 5%-Lutrol	0.66 ± 0.00	17.82 ± 0.35	0.999
PEG 10%-Lutrol	0.65 ± 0.03	53.43 ± 7.17	0.993
PEG 15%-Lutrol	0.61 ± 0.05	10.9 6 ± 0.39	0.993

**Table 6:** Ex vivo permeation pharmacokinetic parameters (n: release exponent).

**Corneal Permeation**			
**Formulation**	**Best Fit**	**Pharmacokinetic Parameters**	
PLGA Lutrol	Korsmeyer-Peppas	K: 0.814 ± 0.075	n: 0.33 ± 0.02
PLGA 5% Lutrol	Korsmeyer-Peppas	K: 1.45 ± 0.34	n: 0.40 ± 0.04
PLGA 10% Tween	Hyperbola	K_d_: 38.01 ± 3.64 min	B _max:_ 19.8% ± 0.4904%
PLGA 15% Lutrol	Hyperbola	K_d_: 46.02 ± 4.15 min	B _max_ 27.98% ± 0.7002%
**Scleral Permeation**			
**Formulation**	**Best Fit**	**Pharmacokinetic Parameters**	
PLGA Lutrol	Korsmeyer-Peppas	K: 7.29 ± 0.58	N: 0.14 ± 0.16
PLGA 5% Lutrol	Hyperbola	K_d_: 90.92 ± 8.80 min	B_max:_ 24.64% ± 0.85%
PLGA 10% Tween	Hyperbola	K_d_: 24.26 ± 3.90 min	B_max:_ 21.57% ± 0.75%
PLGA 15% Lutrol	Hyperbola	K_d_: 19.23 ± 2.59 min	B_max:_ 23.05% ± 0.61%
